# Supplementary material for: Redefining the role of AMPK in autophagy and the energy stress response
Source: Nat Commun. 2023 May 24;14:2994. doi: 10.1038/s41467-023-38401-z (PMC10209092; doi:10.1038/s41467-023-38401-z)
Supplement: Supplementary file 1 — Supplementary Information [file 41467_2023_38401_MOESM1_ESM.pdf]

Supplementary Information

Redefining the role of AMPK in autophagy and the energy stress response

Ji-Man Park[1], Da-Hye Lee[1], and Do-Hyung Kim[1,2,3,4]

[1]Department of Biochemistry, Molecular Biology and Biophysics, University of Minnesota, Twin Cities, Minneapolis, MN, 55455, USA.

[2]Institute for Diabetes, Obesity and Metabolism, University of Minnesota, Twin Cities, Minneapolis, MN, USA.

[3]Center for Immunology, University of Minnesota, Twin Cities, Minneapolis, MN, 55455, USA.

[4]Masonic Cancer Center, University of Minnesota, Twin Cities, Minneapolis, MN, 55455, USA.

List of Supplementary Figures

Supplementary Fig. 1. AMPK inhibits ULK1 signaling to the autophagy initiation machinery.
Supplementary Fig. 2. LKB1-AMPK axis inhibits ULK1-autophagy signaling during energy stress.
Supplementary Fig. 3. AMPK inhibits autophagy induction but is required for optimal autophagy.
Supplementary Fig. 4. AMPK stabilizes its binding with ULK1 by phosphorylating ULK1 at Ser556 and Thr660.
Supplementary Fig. 5. AMPK inhibits ULK1 activity by phosphorylating ULK1 at Ser556 and Thr660.
Supplementary Fig. 6. AMPK suppresses Atg14-associated Vps34 and autophagy by phosphorylating ULK1 at Ser556 and Thr660.
Supplementary Fig. 7. The AMPK-ULK1 pathway enables cells to maintain autophagy capability and extends cell survival during glucose starvation.

List of Supplementary Tables

Supplementary Table 1. Primers used for site-directed mutagenesis.
Supplementary Table 2. Sequences of gRNA used for developing knockout cell lines using CRISPR-cas9 genome editing tools.
Supplementary Table 3. Primers used for qPCR analysis of gene expression.

Supplementary Figure 1

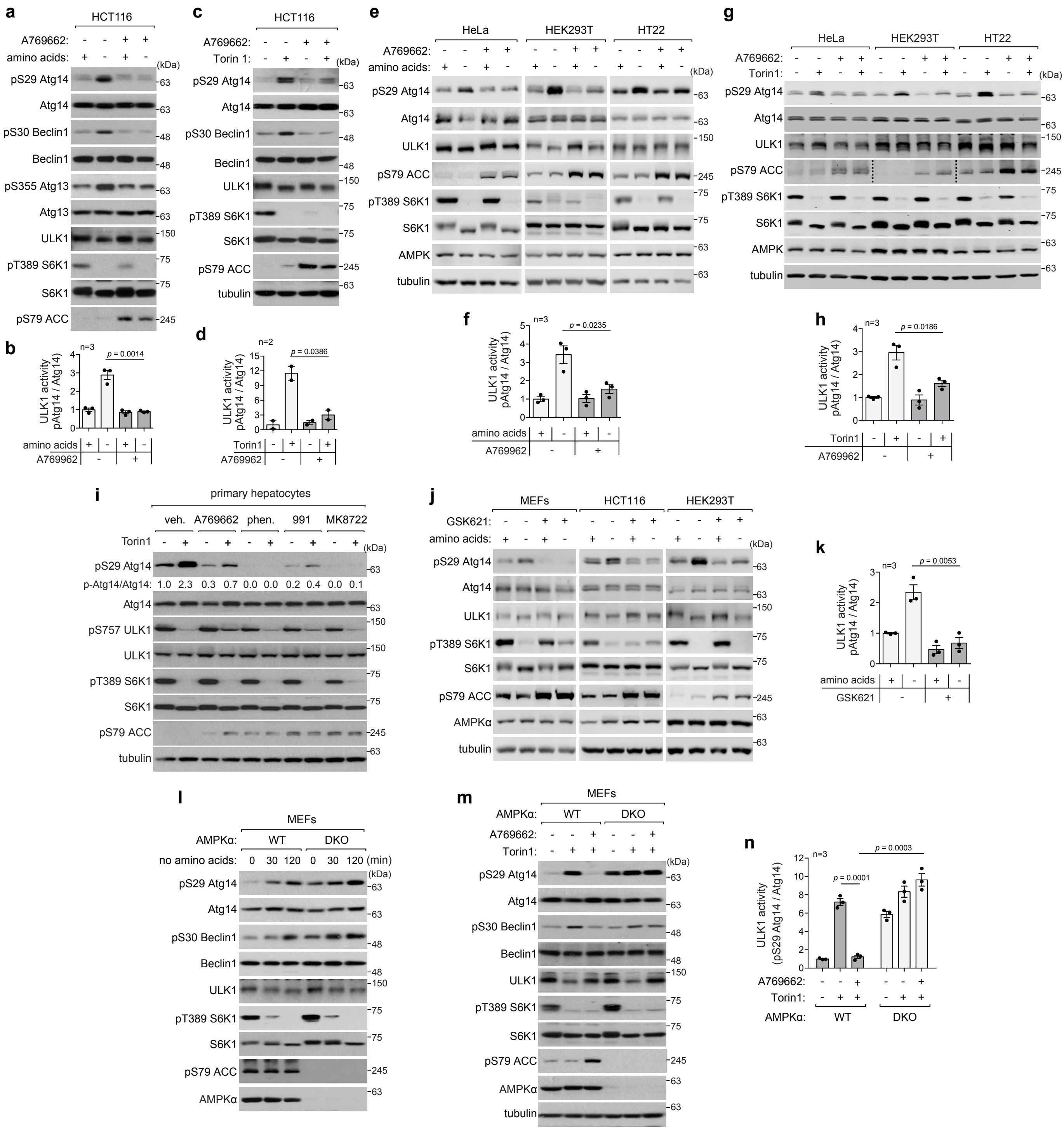

Supplementary Figure 1, continued

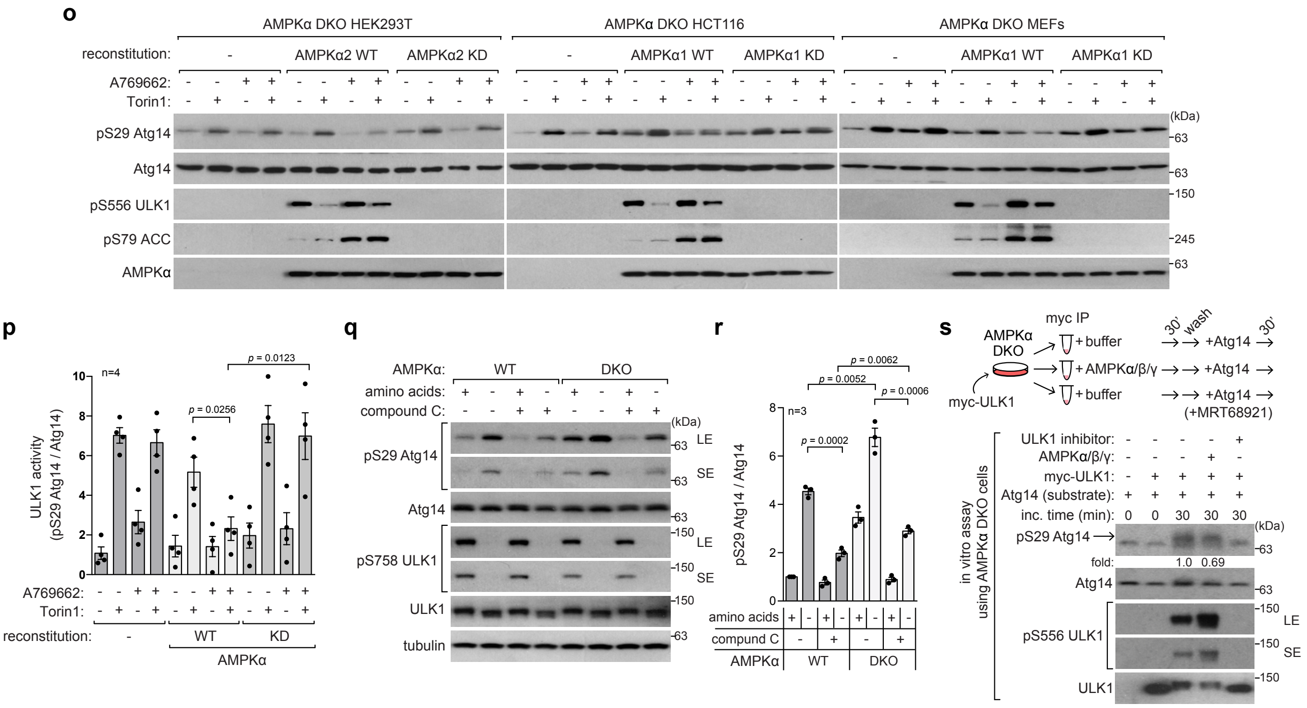

Supplementary Figure 1. **AMPK inhibits ULK1 signaling to the autophagy initiation machinery. a-d** AMPK activation suppresses mTORC1 inhibition-induced activation of ULK1. HCT116 cells were treated with either DMSO (-), the vehicle control, or A769662 (+) for 30 min, followed by incubation in either full or amino acid-deprived medium for 2 h or treated with either the vehicle or Torin1 (250 nM) for 1 h. **e** A769662 (100 μM) suppresses amino acid starvation-mediated increase in ULK1 activity in HeLa, HEK293T and HT22 cells. Cells were treated as described in (**a**). **f** Quantitation of ULK1 activity from (**e**). The measurement considers the three cell lines. **g** A769662 suppresses Torin1-mediated increase in ULK1 activity in HeLa, HEK293T and HT22 cells. Cells were treated as described in (**c**). **h** Quantitation of ULK1 activity from (**g**). The measurement considers the three cell lines. **i** AMPK activation suppresses ULK1 activation in primary hepatocytes. Mouse primary hepatocytes were treated with vehicle (DMSO), A769662 (100 μM), phenformin (2 mM), 991 (10 μM) or MK8722 (10 μM) for 30 min, followed by a 1-h treatment with Torin1. **j** GSK621, a potent allosteric activator of AMPK, suppresses the amino acid starvation-induced increase in ULK1 activity. Cells were treated with GSK621 (10 μM) for 30 min, followed by incubation in either full or amino acid-deprived medium for 1 h. **k** Quantitation of ULK1 activity from (**j**). The measurement considers the three cell lines. **l** Depletion of AMPKα led to an increase in ULK1 activity and a reduction in ULK1 responsiveness to amino acid starvation. AMPKα WT and DKO MEFs were starved of amino acids for the indicated duration. **m, n** The inhibitory effect of A769662 on ULK1 activity depends on AMPK. AMPKα WT and DKO MEFs were treated as described in (**c**). **o** Reconstitution of WT AMPKα, but not the KD mutant, restored the ability of A769662 to inhibit ULK1 activity in AMPKα DKO cells. The WT or the KD form of AMPKα1 or AMPKα2 was stably expressed in AMPKα DKO HEK293T, HCT116 and MEF cells. The cells were treated with vehicle (-) or A769662 at 100 μM (+) for 30 min, followed by treatment with Torin1 (250 nM) for 1 h. **p** Quantitative analysis of ULK1 activity from (**o**) and Figure 1l. **q, r** Compound C suppresses ULK1 activity independently of AMPK. AMPKα WT and DKO HCT116 cells were treated with compound C (10 μM) for 30 min, then incubated in either full or amino acid-deprived medium for 1 h (LE: long exposure; SE: short exposure). **s** In vitro kinase assay for ULK1 shows that AMPK moderately reduces ULK1-mediated phosphorylation of Atg14 Ser29. However, ULK1 autophosphorylates Ser556 in the absence of AMPK in vitro. The values in the graphs of this figure are represented as the mean ± SEM. Statistical analysis was conducted by two tailed Student t-test. Further information on the statistical analysis can be found in the "Statistics and Reproducibility" section of the Methods. The number of independent experiments is indicated by the 'n' value on each graph. Source data are provided as a Source Data file.

Supplementary Figure 2

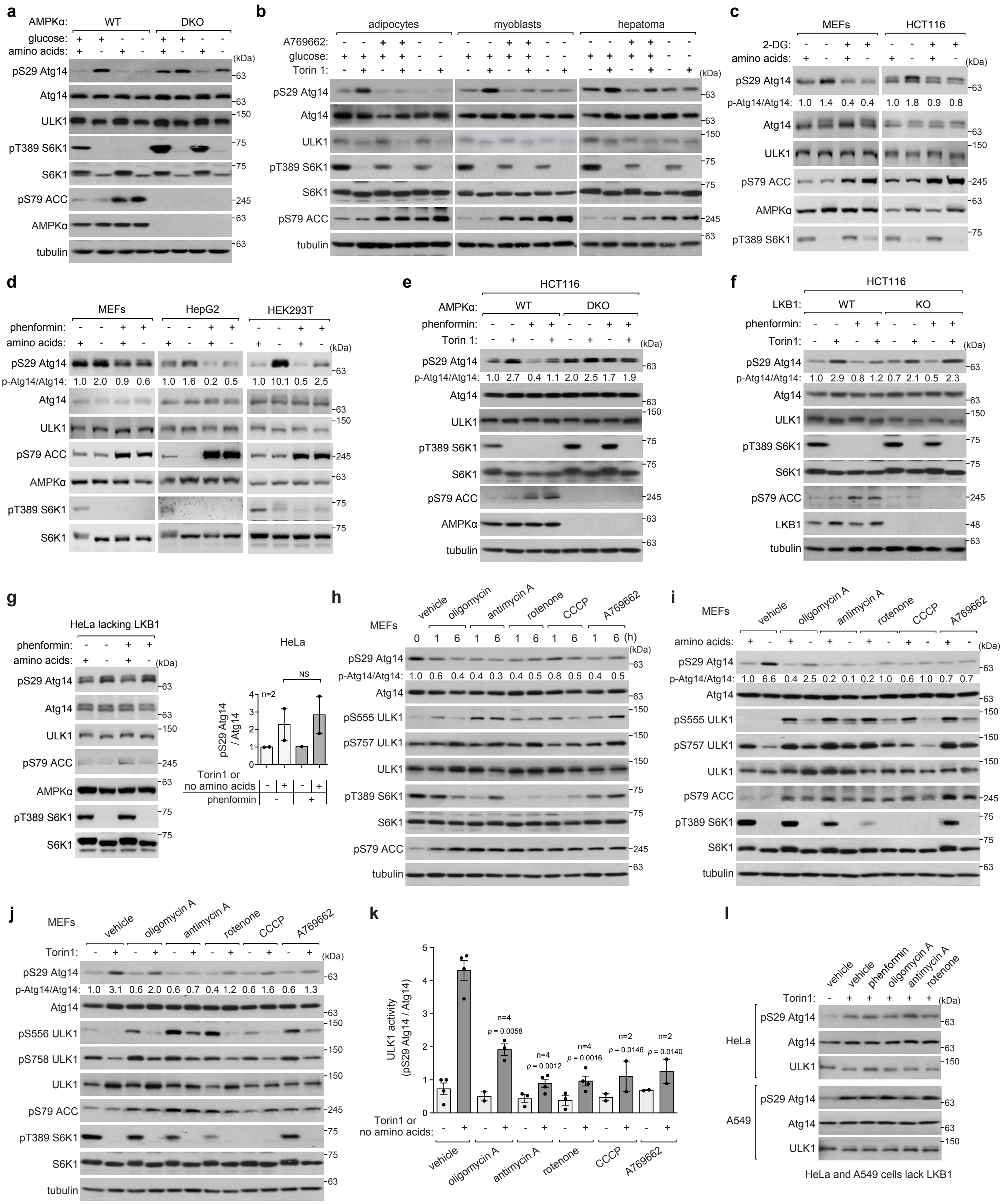

Supplementary Figure 2. LKB1-AMPK axis inhibits ULK1-autophagy signaling during energy stress. **a** Glucose starvation suppresses amino acid starvation-induced activation of ULK1 via AMPK. AMPKα WT and DKO MEFs were starved of glucose for 2 h, then starved of amino acids for 2 h while maintaining the same glucose condition. **b** Glucose starvation or A769662 suppresses ULK1 activation in response to Torin1 in 3T3-L1 adipocytes, C2C12 myoblasts, and HepG2 hepatoma cells. The cells were treated with A769662 (100 μM) or starved of glucose for 2 h, then treated with Torin1 (250 nM) for 1 h. **c** Treatment with 2-DG suppresses amino acid starvation-induced activation of ULK1. MEFs and HCT116 cells were treated with 2-DG (10 mM) for 1 h, then incubated in either full (+) or amino acid-deprived medium (-) for 1 h. The 2-DG treatment was maintained at a constant level throughout the entire period of amino acid starvation. **d** Phenformin suppresses amino acid starvation-induced activation of ULK1 in MEFs, HepG2, and HEK293T cells. The cells were treated with phenformin (2 mM) for 2 h, then incubated in full or amino acid-deprived medium for 1 h. Phenformin was maintained at the same level throughout the entire period of amino acid starvation. **e** Phenformin suppresses Torin1-induced activation of ULK1 via AMPK. AMPKα WT and DKO HCT116 cells were treated with phenformin (2 mM) for 2 h, then treated with Torin1 (250 nM) for 1 h. **f** The suppressive effect of phenformin on ULK1 activity depends on LKB1. LKB1 WT or KO MEFs were pre-treated with phenformin (2 mM) for 2 h then treated with Torin1 for 1 h. **g** Phenformin does not suppress amino acid starvation-induced activation of ULK1 in HeLa cells that naturally lack LKB1. The cells were pre-treated with phenformin (2 mM) for 2 h, then incubated in either full or amino acid-deprived medium for 1 h. Phenformin remained the same during amino acid starvation. **h** Mitochondrial inhibitors suppress ULK1 activity. MEFs were treated with vehicle (-), mitochondrial inhibitors, or A769662 for the specified periods. The concentrations used for each treatment were 10 μM for oligomycin A and antimycin A; 1 μM for rotenone, 25 μM for CCCP, 100 μM for A769662. **i** Mitochondrial inhibitors suppress amino acid starvation-induced activation of ULK1. MEFs were treated with the indicated mitochondrial inhibitors or A769662 for 30 min, then incubated in either full or amino acid-deprived medium for 2 h. **j** Mitochondrial inhibitors suppress Torin1-induced activation of ULK1. MEFs were treated with vehicle, mitochondrial inhibitors, or A769662 for 30 min, then treated with Torin1 (250 nM) for 1 h. **k** Quantitative analysis of ULK1 activity from (**i**) and (**j**). **l** HeLa and A549 cells, which are deficient of LKB1, did not exhibit the suppression of Torin1-induced ULK1 activation by phenformin and mitochondrial inhibitors. The cells were treated as described in (**j**). The statistical analysis in this figure was performed as described in Supplementary Figure 1. The p values on the graph of (**k**) are in comparison to the vehicle control with Torin1 treatment or amino acid starvation. The “NS” on the graph of (**g**) indicates that the difference is not statistically significant. The number of independent experiments is indicated by the ‘n’ value on each graph. Source data are provided as a Source Data file.

Supplementary Figure 3

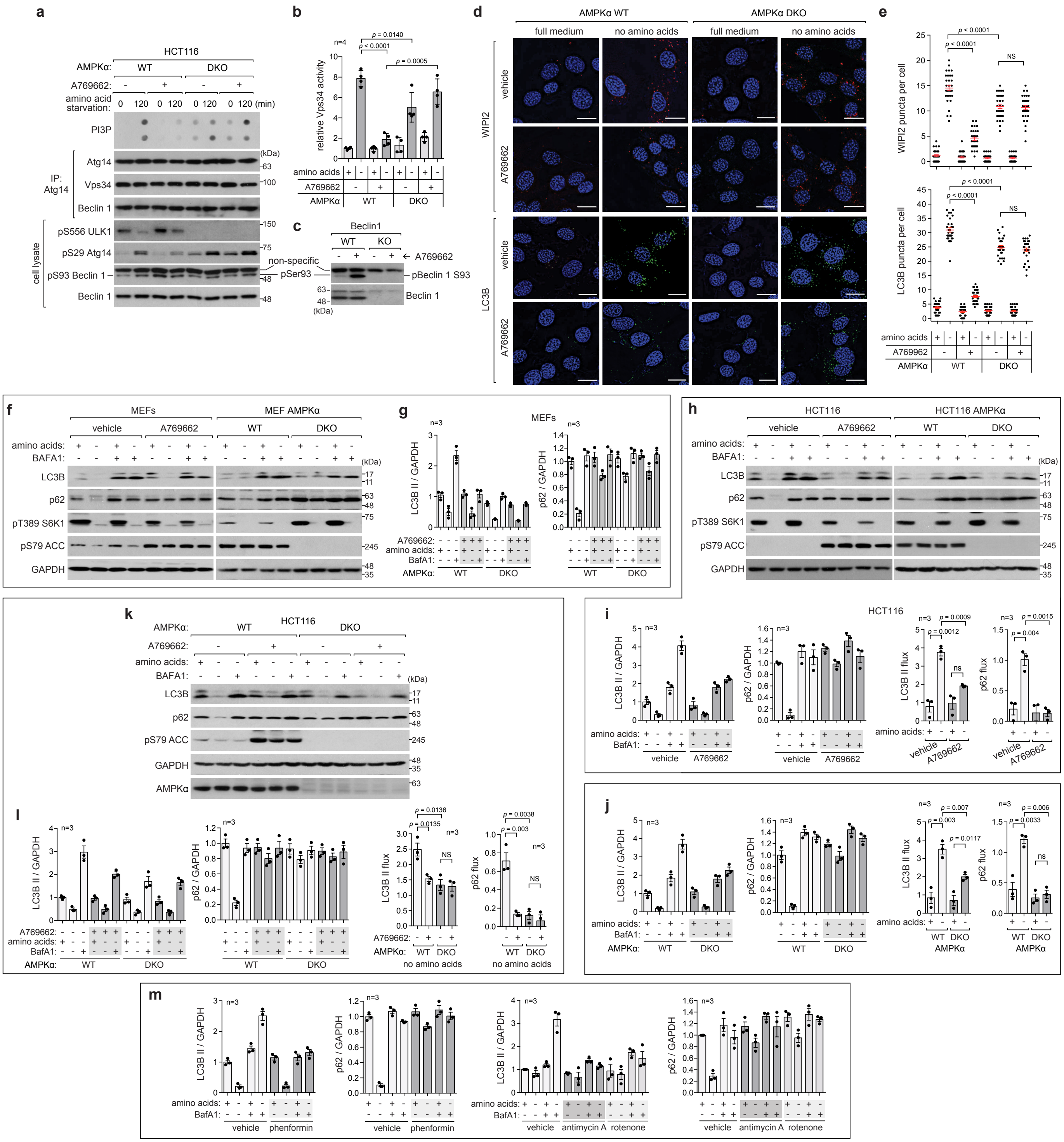

Supplementary Figure 3, continued

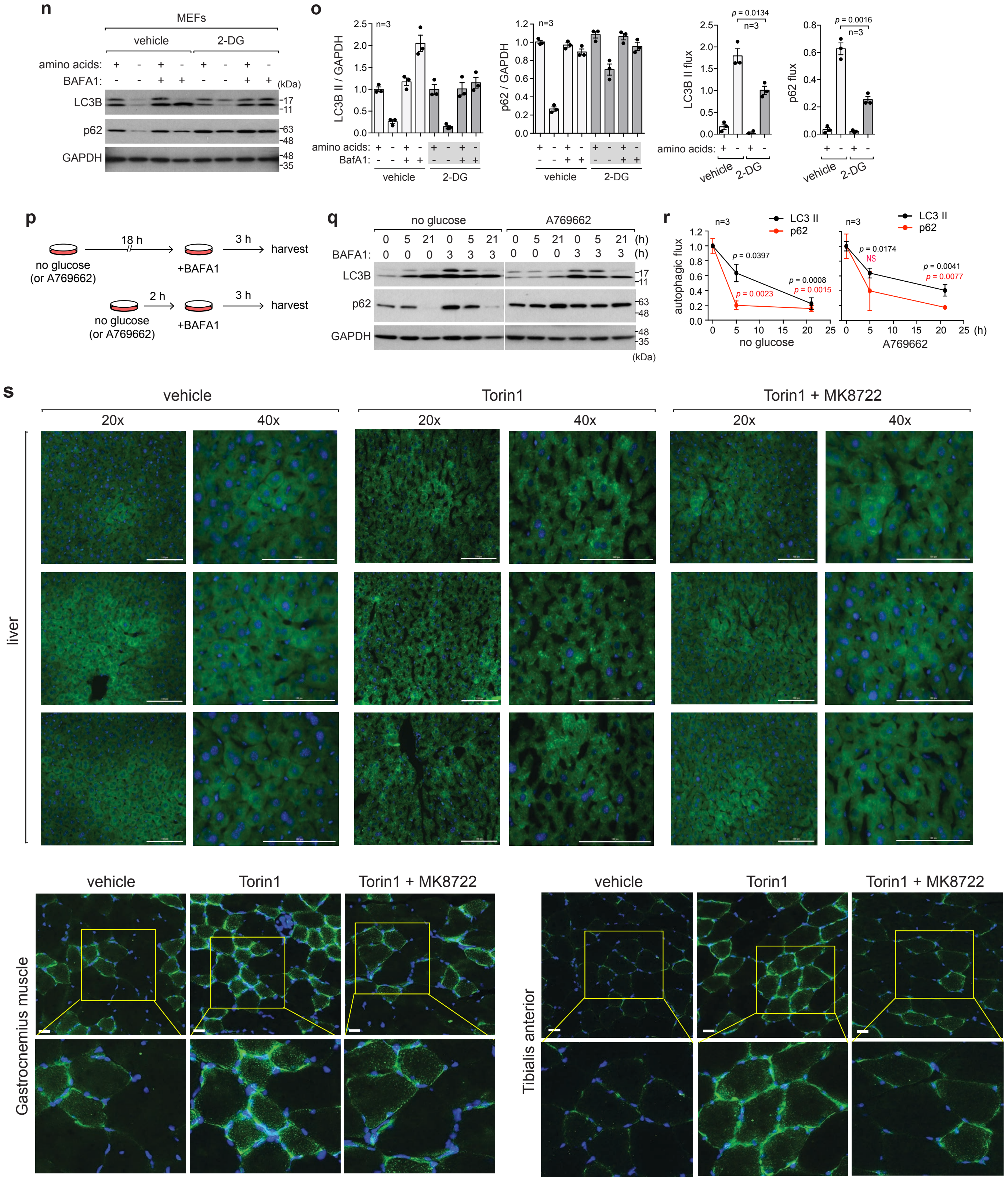

Supplementary Figure 3, continued

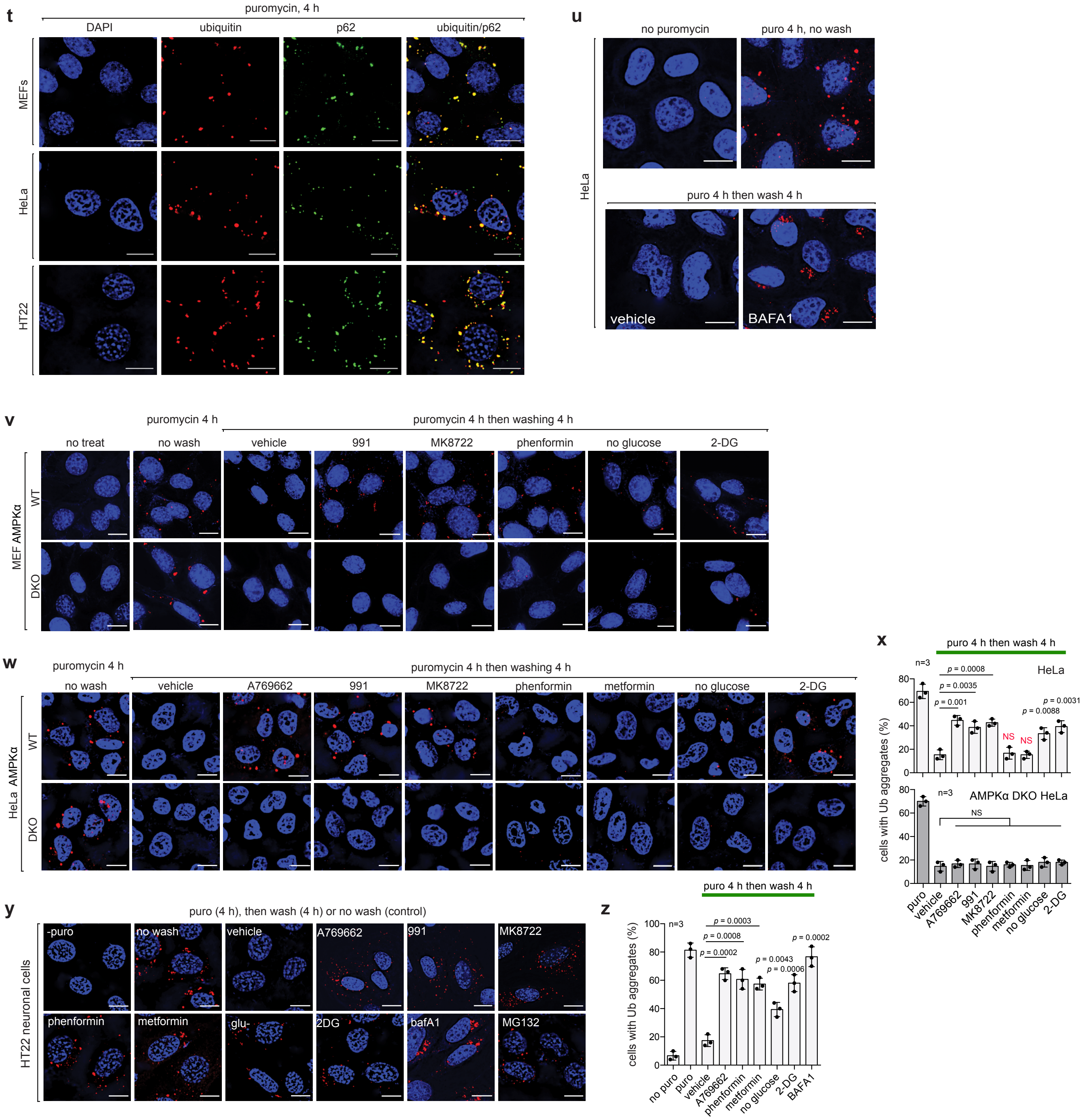

Supplementary Figure 3. AMPK inhibits autophagy induction but supports optimal autophagy. a AMPK activation suppresses amino acid starvation-induced activation of the Atg14-associated Vps34 complex. AMPKα WT and DKO HCT116 cells were treated with vehicle (-) or 100 μM of A769662 (+) for 30 min, then incubated in either full or amino acid-deprived medium for 2 h. Vps34 activity was assessed in vitro using anti-Atg14 immunoprecipitates isolated from the cells and phosphatidylinositol (PI) as substrate. PI3P, the product of the reaction, was analyzed by a dot blot assay as described in Methods. **b** Quantitative analysis of Vps34 activity from (**a**). The y-axis values represent the relative levels of PI3P compared to those obtained from WT cells treated with the vehicle in complete medium. **c** Confirmation of

the specific band for Beclin 1 Ser93 (mouse Ser91) phosphorylation. Beclin 1 WT and KO HCT116 cells were treated with A769662 (100 μM) for 1 h. **d** AMPK activation suppresses the formation of phagophores and autophagosomes during amino acid starvation. AMPKα WT and DKO MEFs were treated with either vehicle or A769662 for 30 min, then incubated in either full or amino acid-deprived medium for 2 h. Immunostaining was conducted to detect phagophores and autophagosomes using anti-WIPI2 (red) and anti-LC3B (green) antibodies, respectively. **e** Quantitative analysis of (**d**). A total of more than 30 cells were analyzed across three independent experiments. **f-j** AMPK activation and AMPKα deficiency suppress autophagy flux. The indicated cells were incubated in either full or amino acid-deprived medium for 4 h, with or without the addition of A769662 (100 μM) and BAFA1 (200 nM). The values for the flux in the graphs represent the difference in LC3B II or p62 levels between the conditions with and without BAFA1. **k, l** A769662 suppresses autophagy flux through AMPK activation. The cells were treated as described in (**f**). **m** Phenformin and mitochondrial inhibitors suppress autophagy flux during amino acid starvation. MEFs were starved of amino acids in the presence of the vehicle or the chemicals for 4 h. The blot data are presented in Figure 3h. **n, o** 2-DG suppresses autophagy flux during amino acid starvation. MEFs were treated with 2-DG (20 mM) for 4 h, under conditions either with or without amino acids and BAFA1. **p** Diagram depicting the experimental design to analyze the effect of prolonged glucose starvation and A769662 treatment on autophagy flux. HCT116 cells were starved of glucose or treated with A769662 (100 μM) during the indicated hours. To prevent toxicity associated with extended BAFA1 treatment, the drug was only administered during the final three hours of incubation before the cells were harvested for analysis. **q, r** Glucose starvation and A769662 suppress basal autophagy flux. The values in the graphs represent the relative levels of the proteins at the specified time points compared to their levels at 0 h. **s** AMPK activation inhibits Torin1-induced autophagy in the mouse liver and gastrocnemius and tibialis anterior skeletal muscle. The mouse was treated as described in Figure 1m and Methods. LC3B puncta were analyzed in liver cells and muscle fibers by staining with anti-LC3B antibody. **t** Puromycin treatment leads to the formation of aggresomes. MEF, HeLa, and HT22 cells were treated with puromycin (5 μM) for 4 h. Ubiquitin and p62 were detected using anti-ubiquitin (red) and anti-p62 (green) antibodies by immunostaining. For an unknown reason, the treatment of HCT116 cells with puromycin failed to trigger the formation of aggresomes. **u** Confirmation of the clearance of aggresomes by aggrephagy. HeLa cells were treated with puromycin as described in (**t**), then either fixed for staining (no wash) or incubated in full medium without puromycin (wash) for an additional 4 h in the presence or absence of BAFA1 (200 nM). Ubiquitinated protein aggregates (red) were analyzed by immunostaining as in described in (**t**). **v** AMPK activation suppresses aggrephagy in MEFs. The cells were treated with puromycin for 4 h then either fixed for staining (no wash) or incubated in full medium in the presence of either vehicle or one of the following agents: 991 (10 μM), MK8722 (10 μM), phenformin (2 mM), 2-DG (20 mM), or in glucose-deprived medium. This incubation was performed for an additional 4 h in the absence of puromycin before the cells were fixed for analysis. A selection of the images is also presented in Figure 3p. **w** AMPK activation suppresses aggrephagy in HeLa cells. AMPKα WT and DKO HeLa were treated as described in (**v**), with A769662 and metformin added at 100 μM and 10 mM, respectively. **x** A quantitative analysis was performed to assess the effects of AMPK activating agents on aggrephagy in AMPKα WT and DKO HeLa cells. The results are displayed as a percentage of cells with red puncta above a threshold, as determined using ImageJ. The data was collected from a total of 50 cells across three independent experiments. **y, z** AMPK activation suppresses aggrephagy in HT22 neuronal cells. HT22 cells were treated as described above. In the cell and tissues images in this figure, nuclei were stained with DAPI (blue). A scale bar of 10 μm is for cell images and 25 μm for tissue images. The statistical analysis in this figure was performed as described in Supplementary Figure 1 and Methods. The number of independent experiments is indicated by the 'n' value on each graph. Source data are provided as a Source Data file.

Supplementary Figure 4

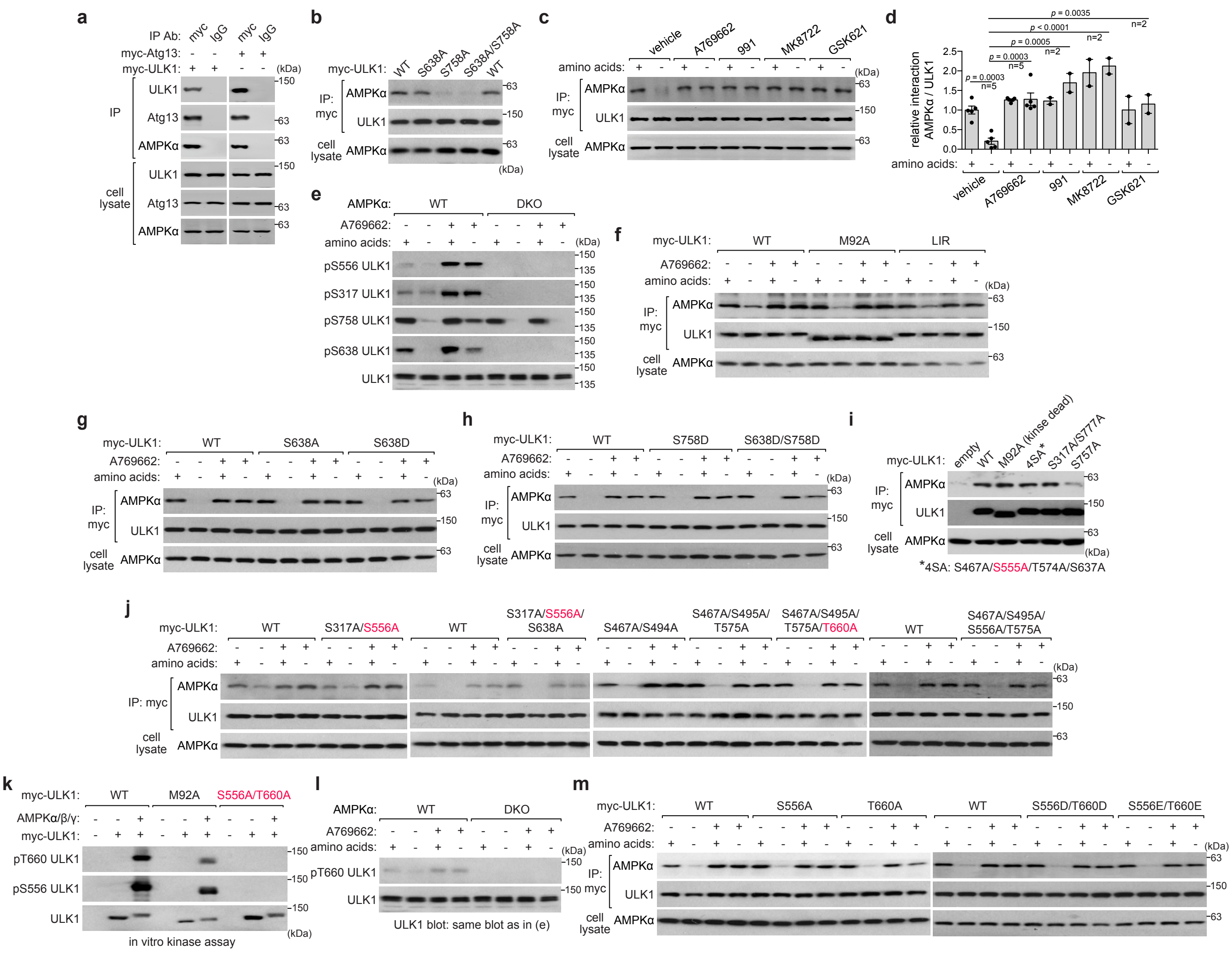

Supplementary Figure 4. AMPK stabilizes its binding with ULK1 by phosphorylating ULK1 at Ser556 and Thr660. a Confirmation of the specificity of interaction between AMPK and ULK1 detected by the co-immunoprecipitation assay. ULK1 KO HEK293T cells (left set) and the original HEK293T cells (right set) were transiently transfected with myc-tagged ULK1 and Atg13, respectively. The endogenous proteins were isolated via anti-myc immunoprecipitation and analyzed by western blotting. **b** The ULK1 S758A mutation disrupts the AMPK-ULK1 interaction. ULK1 KO HEK293T cells were transiently transfected with myc-tagged ULK1 constructs, and two days post-transduction, myc-ULK1 immunoprecipitates were isolated using anti anti-myc antibody. The amount of endogenous AMPKα bound to the immunoprecipitates was analyzed by western blotting. **c, d** AMPK activation stabilizes the AMPK-ULK1 interaction. ULK1 KO HEK293T cells that were transduced with myc-ULK1 were incubated in either full or amino acid-deprived medium in the presence of the vehicle or the chemicals for 1 h. The concentrations of the chemicals used for this experiment are the same as those described in Figure 1. The statistical analysis was performed as described in Supplementary Figure 1. The number of independent experiments is indicated by the 'n' value on the graph. **e** AMPK activation stabilizes ULK1 phosphorylation at Ser758 and Ser638, which are targeted by mTORC1, under amino acid starvation. AMPKα WT and DKO HCT116 cells were incubated in either full or amino acid-deprived medium in the presence of the vehicle or A769662 (100 μM) for 1 h. **f** The stabilizing effect of A769662 on the AMPK-ULK1 interaction is not dependent on ULK1 kinase activity or binding to Atg8. ULK1 KO HEK293T cells were transiently transfected with WT

ULK1, a kinase dead mutant (M92A), or a mutant that is unable to bind to Atg8 (LIR). Two days after transfection, the cells were treated and the interaction between AMPK and ULK1 was analyzed as described in (**b**) and (**c**). **g** The AMPK-ULK1 interaction does not depend on ULK1 Ser638 phosphorylation. ULK1 KO HEK293T cells transduced with the indicated ULK1 constructs were treated and analyzed for the interaction as described in (**b**) and (**c**). **h** The ULK1 S758D mutation does not enhance the stability of the AMPK-ULK1 interaction during amino acid starvation. **i** Mutating mouse ULK1 Ser317, Ser467, Ser556, Thr574, Ser637, and Ser777 with alanine does not alter the AMPK-ULK1 interaction. ULK1 KO HEK293T cells transiently transfected with the indicated constructs were used for analysis of interaction. The cells were cultured in a full growth medium. **j** ULK1 mutants that do not contain both S556A and T660A behave similarly to WT ULK1 in their interaction with AMPK in response to A769662. ULK1 KO HEK293T cells transiently transfected with the indicated constructs were used for analysis of interaction as described in (**b**) and (**c**). **k** AMPK directly phosphorylates ULK1 Thr660. Myc-tagged WT or mutant ULK1 was transiently expressed in HEK293T cells. Two days after transduction, anti-myc immunoprecipitates were isolated and incubated with recombinant AMPKα1/β1/γ1 complex in the presence of both ATP and AMP. The phosphorylation status of ULK1 was assessed using antibodies specific to Ser556 and Thr660 phosphorylation sites. **l** The phosphorylation of ULK1 at Thr660 in cells depends on AMPK activity. AMPKα WT and DKO HCT116 cells were treated with A769662 (100 μM) for 30 min and then incubated in either full or amino acid-deprived medium for 1 h. **m** Replacing either Ser556 or Thr660 with alanine does not abolish the stabilizing effect of A769662 on the AMPK-ULK1 interaction, and replacing both residues with aspartate or glutamate does not enhance the stability of the AMPK-ULK1 interaction during amino acid starvation. Source data are provided as a Source Data file.

Supplementary Figure 5

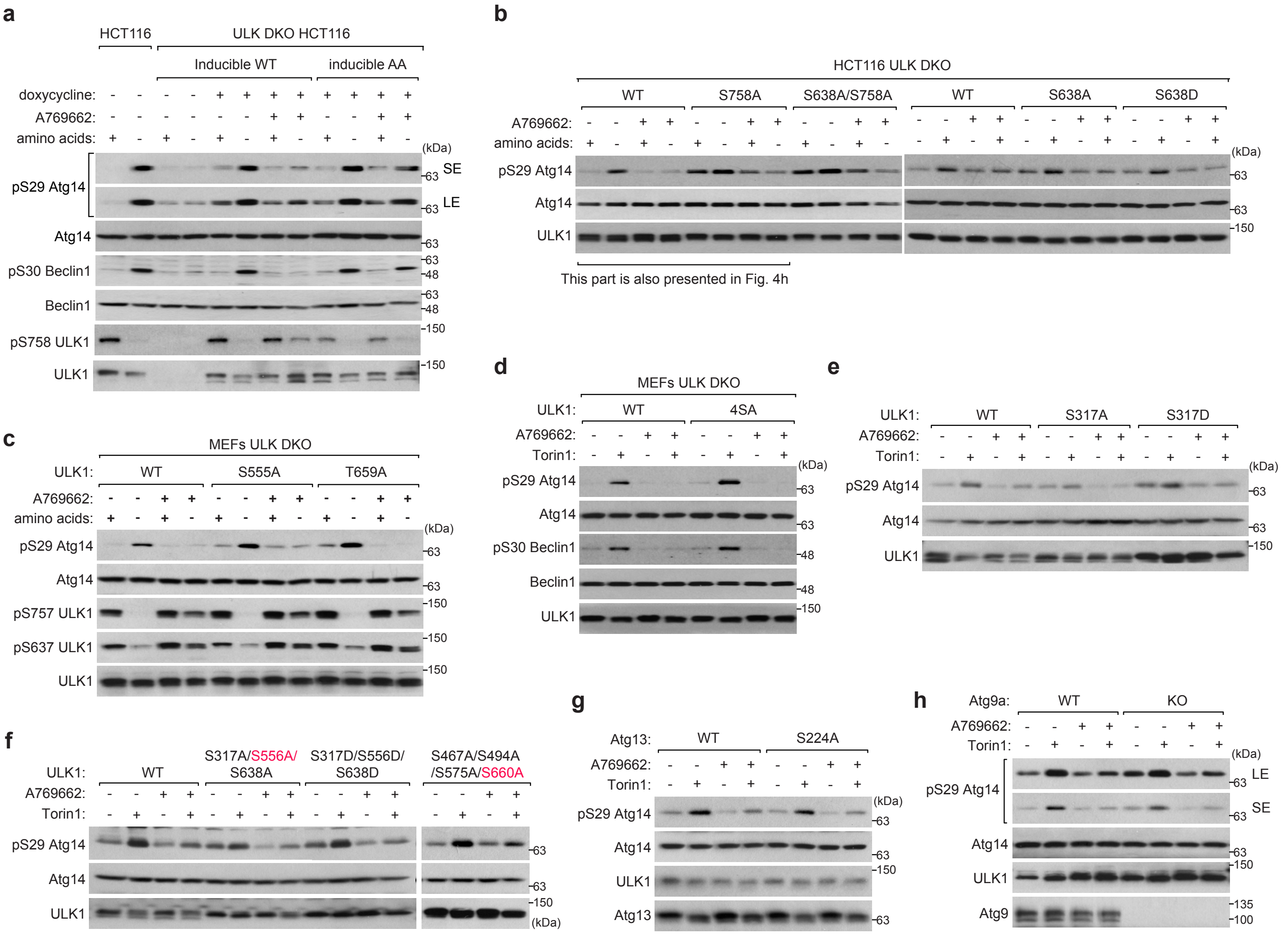

Supplementary Figure 5. AMPK inhibits ULK1 activity by phosphorylating ULK1 at Ser556 and Thr660. **a** The ability of AMPK to suppress ULK1 activity requires the phosphorylation of ULK1 at either Ser556 or Thr660. ULK DKO HCT116 cells were stably transduced with a WT or AA construct, which is controlled by a doxycycline-inducible promoter. After a 16-h treatment with doxycycline (100 ng/ml) to induce the expression of ULK1 to a level equivalent to the endogenous ULK1 level, the cells were incubated in either full or amino acid-deprived medium, with or without A769662 (100 μM), for 1 h. (SE: short exposure; LE: long exposure). **b** ULK1 Ser758 phosphorylation is crucial to suppress ULK1 activity. ULK DKO HCT116 cells stably transduced with WT or mutant ULK1 were treated and analyzed as in (**a**). **c** Replacing either Ser556 or Thr660 with alanine does not abolish the suppressive effect of A769662 on ULK1 activity. ULK DKO MEFs were stably transduced with mouse WT or mutant ULK1. The cells were treated and analyzed as in described in (**a**). **d** The 4SA mutant of mouse ULK1, which contains S467A, S556A, T574A, and S637A mutations, exhibits similar activity to WT ULK1 in response to AMPK activation. ULK DKO MEFs stably transduced with either WT ULK1 or the 4SA mutant were treated with A769662 (100 μM) and/or Torin1 (250 nM) for 1 h. **e, f** The activity of ULK1 mutants except those with both S556A and T660A mutations is similar to that of WT ULK1 in response to AMPK activation. ULK DKO HCT116 were stably transduced with the indicated ULK1 constructs and treated as in (**d**). **g** The phosphorylation of Atg13 at Ser224 does not affect the ability of A769662 to suppress ULK1 activity. Atg13 KO HEK293T cells were stably transduced with either the WT or S224A Atg13 mutant. The transduced cells were treated and analyzed as described in (**d**). **h** The presence or absence of Atg9a does not affect the AMPK-mediated suppression of ULK1 activity. WT and Atg9a KO MEFs were treated as indicated and analyzed for ULK1 activity as described in (**d**). Source data are provided as a Source Data file.

Supplementary Figure 6

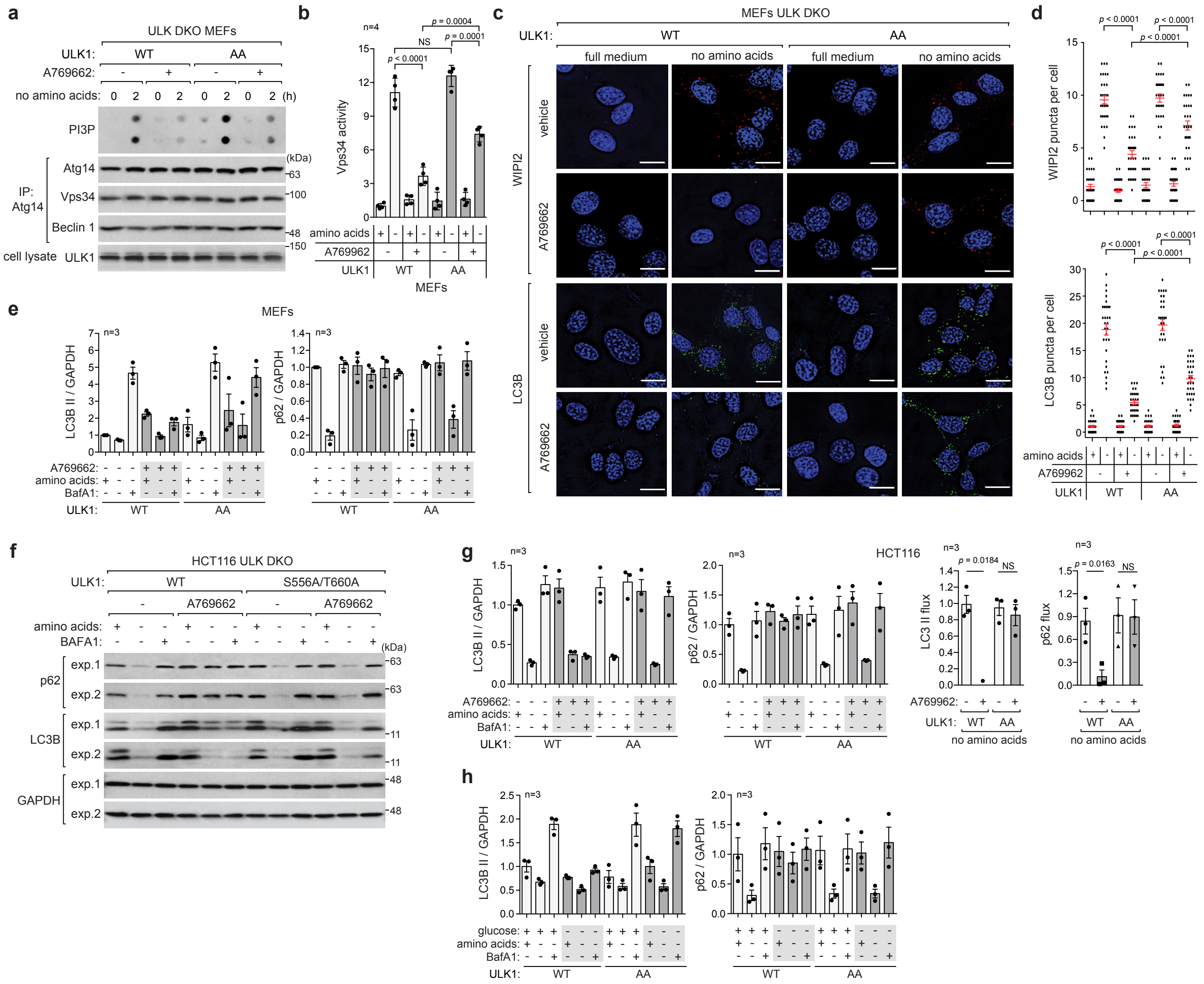

Supplementary Figure 6. AMPK suppresses Atg14-associated Vps34 activity and autophagy by phosphorylating ULK1 at Ser556 and Thr660. a The AMPK-mediated phosphorylations of ULK1 at Ser556 and Thr660 suppress the activity of Atg14-associated Vps34. ULK DKO MEFs stably transduced with either the WT or AA ULK1 construct were incubated in full or amino acid-deprived medium in the presence or absence of A769662 for 2 h. The kinase activity of Atg14-associated Vps34 was analyzed in vitro as described in Methods. **b** Quantitative analysis of Vps34 activity from (**a**). The y-axis values represent the relative levels of PI3P compared to those obtained from WT cells treated with the vehicle in complete medium. **c** ULK1 Ser556 and Thr660 phosphorylations suppress phagophore and autophagosome formation. ULK DKO MEFs stably transduced with WT or AA ULK1 were treated as described in (**a**). Endogenous WIPI2 puncta (red) and endogenous LC3B puncta (green) were stained using anti-WIPI2 antibody and anti-LC3B antibody, respectively. Nuclei were stained with DAPI (blue). A scale bar of 10 μm was used. **d** Quantitative analysis of (**c**). A total of more than 30 cells were analyzed across three independent experiments. **e** The phosphorylation of ULK1 at Ser556 and Thr660 by AMPK suppresses autophagy flux during amino acid starvation. ULK DKO MEFs stably transduced by either WT or AA ULK1 were incubated in full or amino acid-deprived medium in the presence or absence of A769662 and BAFA1 for 4 h. The y-axis values in the graphs represent the relative levels of LC3B II and p62

compared to those in WT cells grown in full medium without any treatment. **f** The inhibitory effect of ULK1 Ser556 and Thr660 phosphorylation on autophagy flux was confirmed using HCT116 cells through an experiment identical to the one described in (**e**). **g** Quantitative analysis of autophagy flux from (**f**). The autophagy flux was measured by the difference in the levels of LC3B II and p62 with and without BAFA1 treatment. **h** Glucose starvation-induced suppression of autophagy flux depends on ULK1 phosphorylation at Ser556 and Thr660 by AMPK. WT and AA HCT116 cells were pre-starved of glucose for 2 h, then analyzed for autophagy flux after a 4 h incubation period. Blot data and flux analysis are presented in Figure 6f. The statistical analysis in this figure was performed as described in Supplementary Figure 1. The number of independent experiments is indicated by the 'n' value on each graph. Source data are provided as a Source Data file.

Supplementary Figure 7

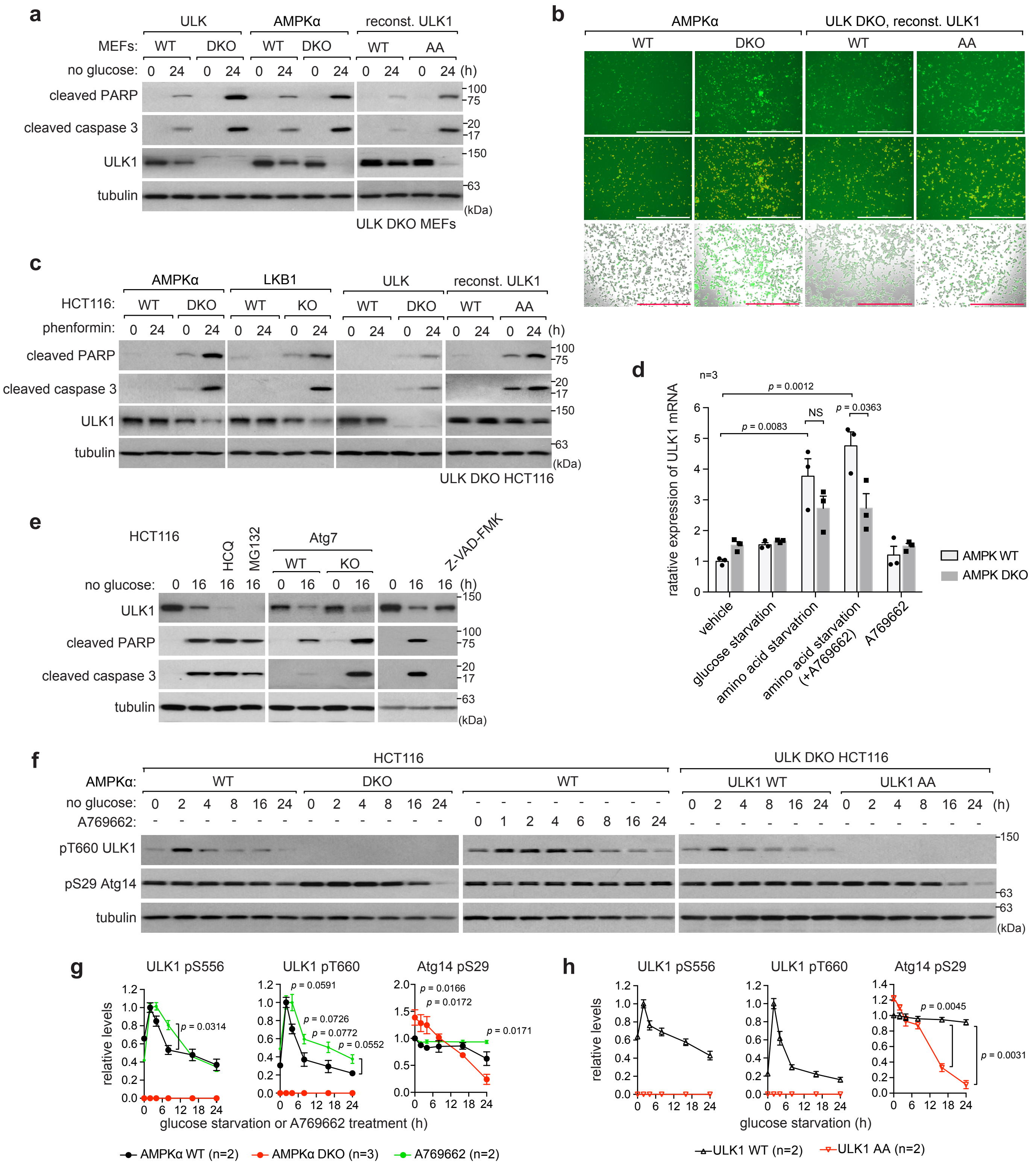

Supplementary Figure 7, continued

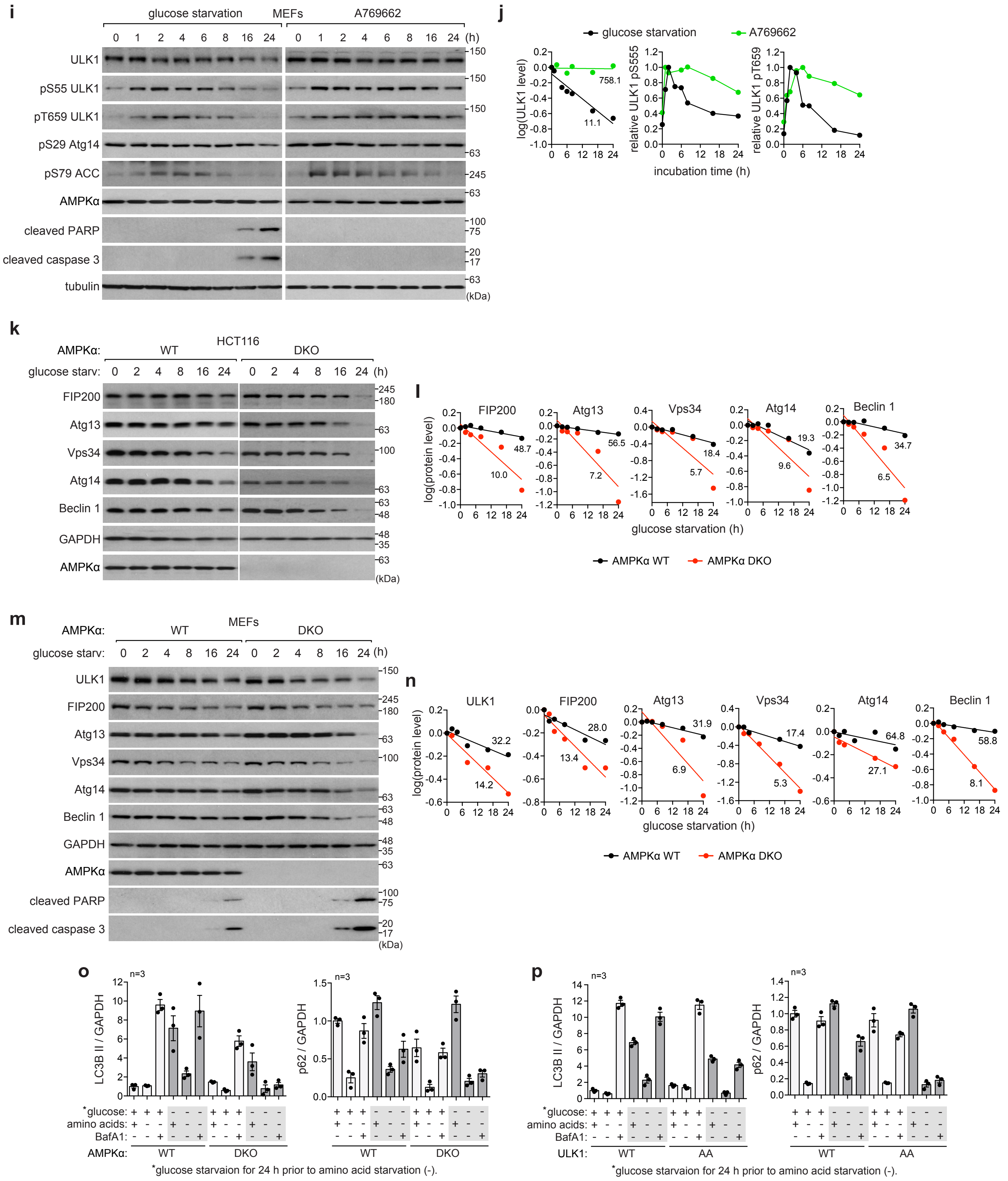

Supplementary Figure 7, continued

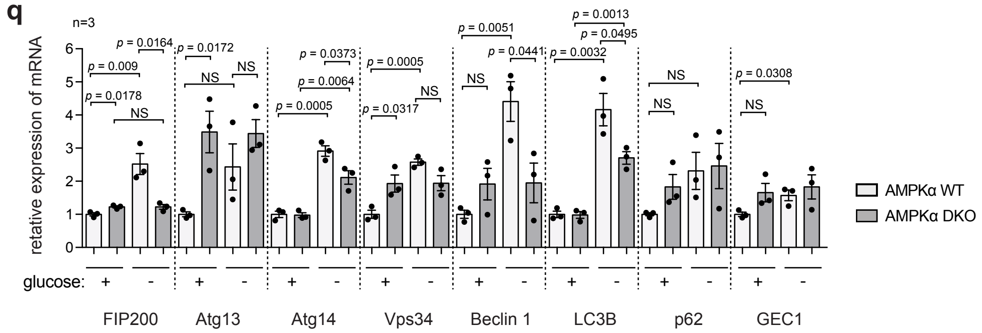

Supplementary Figure 7. The AMPK-ULK1 pathway enables cells to maintain autophagy capability and extends cell survival during glucose starvation. a AMPK-mediated phosphorylation of ULK1 at Ser556 and Thr660 protects cells against apoptosis and prevents ULK1 downregulation during prolonged glucose starvation in MEFs. **b** Staining analysis confirmed that AMPK-mediated phosphorylation of ULK1 at Ser556 and Thr660 plays a protective role in cell survival during prolonged glucose starvation. HCT116 cells were starved of glucose for 0 or 24 h and stained with pSIVA-IANBD (green), a fluorescent probe that labels apoptotic cell membranes. Quantitative analysis of the green fluorescence and bright field images were performed. The cells that were counted were identified by the yellow boundary in the middle row of images. The scale bars represent 1,000 μm. **c** AMPK-mediated phosphorylation of ULK1 at Ser556 and Thr660 protects cells from phenformin-induced apoptosis and prevents the downregulation of ULK1. LKB1 also protects HCT116 cells from phenformin-induced apoptosis and prevents ULK1 downregulation. HCT116 cells were treated with phenformin (1 mM) during the indicated duration. **d** ULK1 expression is not significantly affected by glucose starvation or depletion of AMPKα, and not significantly enhanced by A769662 treatment. AMPKα WT and DKO HCT116 cells were treated with A769662 or starved of glucose or amino acids for 16 h. ULK1 mRNA levels were quantified by qPCR. **e** During prolonged glucose starvation, ULK1 downregulation depends on caspases rather than autophagy or proteasome activity. HCT116 cells were starved of glucose for 16 h in the presence or absence of inhibitors for autophagy (HCQ, 50 μM), proteasome (MG132, 10 μM), or caspases (Z-VAD-FMK, 25 μM). **f** The phosphorylations of ULK1 Thr660 and Atg14 Ser29 were monitored during glucose starvation and A769662 treatment. HCT116 cells were starved of glucose or treated with A769662 (100 μM) for various durations as indicated. **g, h** Quantitative analysis of the indicated phosphorylations during glucose starvation and A769662 treatment. The data were analyzed relative to WT cells or vehicle treatment at the zero-time point. The phosphorylation levels were presented as relative values, with the maximum point normalized to 1. **i** AMPK activation prevents ULK1 from degradation and protects cells against apoptosis during prolonged glucose starvation. MEFs were starved of glucose or treated with A769662 (100 μM) for the indicated hours. **j** Quantitative analysis of ULK1 levels and phosphorylations in (**i**) using tubulin blot as a loading control for normalization. The half-life (hours) of ULK1 in the two conditions is indicated inside the first graph. The phosphorylation levels were relative, with the maximum point set as 1. **k** AMPK stabilizes essential proteins of the autophagy initiation machinery from degradation during prolonged glucose starvation. AMPKα WT and DKO HCT116 cells were starved of glucose for the indicated periods. **l** Quantitative analysis of autophagy proteins in (k) was conducted to determine their half-life under prolonged glucose starvation. The values inside the graphs represent the estimated half-life (hours) of the proteins analyzed. **m** AMPKα WT and DKO MEFs were analyzed similarly as in (**k**), revealing that AMPK prevents the downregulation of key autophagy proteins during prolonged glucose starvation in MEFs. **n** Quantitative analysis of autophagy proteins in (**m**). **o, p** AMPK-mediated phosphorylation of ULK1 at Ser556 and Thr660 is important for the efficient autophagy flux after prolonged glucose starvation. HCT116 cells were starved of glucose for 24 h, followed by a 4-h period for autophagy flux analysis. The y-axis values in the graphs represent the relative levels of LC3B II and p62 compared to those in WT cells grown in full medium without any treatment. **q** AMPKα is important for the upregulation of critical autophagy genes during glucose

starvation. AMPKα WT and DKO HCT116 cells were starved of glucose for 16 h. The mRNAs levels of the indicated genes were quantified by qPCR. The statistical analysis in this figure was performed as described in Supplementary Figure 1. The number of independent experiments is indicated by the 'n' value on each graph. Source data are provided as a Source Data file.

Supplementary Table 1. Primers used for site-directed mutagenesis.

genes	Mutation sites	5'- forward primer sequence -3'<br>5'- reverse primer sequence -3'
Human ULK1	S317A	GCCTCCCCGCCG GCC CTGGGCGAGATG<br>CATCTCGCCCAG GGC CGGCGGGGAGGC
	S467A	CGCAGGTCAGGCGCCACCAGCCCCCTG<br>CAGGGGGCTGGTGGCGCCTGACCTGCG
	S494A	TGGCCAGGAAGATGGCTCTGGGTGGAGGCC<br>GGCCTCCACCCAGAGCCATCTTCCTGGCCA
	S556A	TGCCGCCTGCAC GCT GCCCCCAACCTGTCT<br>AGACAGGTTGGGGGC AGC GTGCAGGCGGCA
	S575A	TGCCCAAACCCCCCGCTGACCCCCTGGGAG<br>CTCCCAGGGGGTCAGCGGGGGGTTTGGGCA
	S638A	TCCCGAAGACCCCCGCCTCCCAGAACCTGCT<br>AGCAGGTTCTGGGAGGCGGGGGTCTTCGGGA
	T660A	CCCTCGAAACCGGGCTCTGCCCGACCTCT<br>AGAGGTCGGGCAGAGCCCGGTTTCGAGGG
	S758A	TCTTCACCGTGGGCGCTCCCCCGAGCGGGA<br>TCCCGCTCGGGGGAGCGCCCACGGTGAAGA
	S556D	TGCCGCCTGCAC GAT GCCCCCAACCTGTCT<br>AGACAGGTTGGGGGC ATC GTGCAGGCGGCA
	S556E	GCCGCCTGCAC GAA GCCCCCAACCTGT<br>ACAGGTTGGGGGCTTCGTGCAGGCGGC
	T660D	CCCTCGAAACCGGGATCTGCCCGACCTCT<br>AGAGGTCGGGCAGATCCCGGTTTCGAGGG
	T660E	CTCGAAACCGGGAACTGCCCGACCTCT<br>AGAGGTCGGGCAGTTCCCGGTTTCGAG
	S638D	TCCCGAAGACCCCCGACTCCCAGAACCTGCT<br>AGCAGGTTCTGGGAGTCGGGGGTCTTCGGGA
	S494D	TGGCCAGGAAGATGGATCTGGGTGGAGGCC<br>GGCCTCCACCCAGATCCATCTTCCTGGCCA
	S758D	TCTTCACCGTGGGCGATCCCCCGAGCGGGA<br>TCCCGCTCGGGGGATCGCCCACGGTGAAGA
Mouse ULK1	S317A	TGGCCTCTCCACCG GCC CTGGGGGAGATG<br>CATCTCCCCCAG GGC CGGTGGAGAGGCCA
	S555A	TGC CGC CTG CAC GCT GCC CCT AAC CTG TC<br>GA CAG GTT AGG GGC AGC GTG CAG GCG GCA
	S637A	TCCCCAAAACCCCCGCCTCTCAGAATTTGCT<br>AGCAAATTCTGAGAGGCGGGGGTTTTGGGGA
	T659A	A CCT CGG AAC CGT GCA CTG CCT GAC CTC T<br>A GAG GTC AGG CAG TGC ACG GTT CCG AGG T
	S757A	TATTTACTGTAGGCGCCCCACCCAGTGGT<br>ACCACTGGGTGGGGCGCCTACAGTAAATA
	S555D	TGC CGC CTG CAC GAT GCC CCT AAC CTG TC<br>GA CAG GTT AGG GGC ATC GTG CAG GCG GCA
	T659D	A CCT CGG AAC CGT GAT CTG CCT GAC CTC TC<br>GA GAG GTC AGG CAG ATC ACG GTT CCG AGG T
	S494D	CTGGCCAGGAAGCTGGACCTTGGAGGTGGCCGT<br>ACGGCCACCTCCAAGGTCCAGCTTCCTGGCCAG
	S757D	GTATTTACTGTAGGCGACCCACCCAGTGGTGCC<br>GGCACCACTGGGTGGGTCGCCTACAGTAAATAC
	S637D	TTCCCCAAAACCCCCGACTCTCAGAATTTGCTG<br>CAGCAAATTCTGAGAGTCGGGGGTTTTGGGGAA
	S467D	ATCCGAAGGTCAGGGGACACCAGCCCCCTGGGC<br>GCCCAGGGGGCTGGTGTCCCCTGACCTTCGGAT
Human Atg13	S224A	CTATCCCAGCTCCGCTCCCATGCACCCCT<br>AGGGGTGCATGGGAGCGGAGCTGGGATAG

Supplementary Table 2. Sequences of gRNA used for developing knockout cell lines using CRISPR-cas9 genome editing tools.

Genes (human)	gRNA sequences 5'- primer sequence -3'	Primers used to confirm the genome editing 5'- forward primer sequence -3' 5'- reverse primer sequence -3'
AMPKα1	TACTCAATCGACAGAAGATT	GATACTCAATCGACAGAAGATT<br>CAGCCTCCCAAAGTACTGGA
AMPKα2	CATACCGAAATCGGCTATCT	TCAGGCTGGTCTCAAACTCC<br>CATACCGAAATCGGCTATCT
ULK1	CGGGCTGACCGATCTTGACG	ACACCATCAGGCTCTTCCTG<br>CGGGCTGACCGATCTTGACG
ULK2	GACCTCGCAGATTATTTGCA	GACCTCGCAGATTATTTGCA<br>ATCGGGTTTCTCCTCTCCTG
LKB1	CAGGTGTCGTCCGCCGCGAA	GTGTGCCTGGACTTCTGTGA<br>AGGTGTCGTCCGCCGCGAA
Atg7	AGAAGAAGCTGAACGAGTAT	AGAAGAAGCTGAACGAGTAT<br>CTGGAGGACCGTGAGGATAA
Atg14	TCTACTTCGACGGCCGCGAC	TCTACTTCGACGGCCGCGAC<br>CTCCTCTCGCTGGTATCTGG

Supplementary Table 3. Primers used for qPCR analysis of gene expression.

Genes (human)	forward primer sequence; 5'- reverse primer sequence
ULK1	5'-GCAAGGACTCTTCCTGTGACAC-3'; 5'-CCACTGCACATCAGGCTGTCTG-3'
LC3B	5'-GAGAAGCAGCTTCCTGTTCTGG-3'; 5'-GTGTCCGTTCACCAACAGGAAG-3'
GEC1	5'-TTGTAGAGAAGGCTCCAAAAGCC-3'; 5'-GGTCTCAGGTGGATTCTCTTCC-3'
p62	5'-TGTGTAGCGTCTGCGAGGGAAA-3'; 5'-AGTGTCCGTGTTTCACCTTCCG-3'
Beclin 1	5'-CTGGACACTCAGCTCAACGTCA-3'; 5'-CTCTAGTGCCAGCTCCTTTAGC-3'
Atg14	5'-TGTACCTGGTCAGTCCAAGCTC-3'; 5'-CAGGTCGGTTTCTTCATCGCTG-3'
Atg13	5'-CAGAACTGCTGGTGAGGACACT-3'; 5'-AGCAGGCTGATAGGAAAGGCGA-3'
Vps34	5'-GCGTTCTTTGCTGGCTGCACAA-3'; 5'-CTCCAAGCAATGCCTGTAGTCTC-3'
ACTB	5'-CACCATTGGCAATGAGCGGTTC-3'; 5'-AGGTCTTTGCGGATGTCCACGT-3'
TBP	5'-TGTATCCACAGTGAATCTTGGTTG-3'; 5'-GGTTCGTGGCTCTCTTATCCTC-3'
