# Supplementary material for: Redefining the role of AMPK in autophagy and the energy stress response
Source: Nat Commun. 2023 May 24;14:2994. doi: 10.1038/s41467-023-38401-z (PMC10209092; doi:10.1038/s41467-023-38401-z)
Supplement: Supplementary file 3 — Reporting Summary [file 41467_2023_38401_MOESM3_ESM.pdf]

nature portfolio

Corresponding author(s): Do-Hyung Kim

Last updated by author(s): Apr 5, 2023

Reporting Summary

Nature Portfolio wishes to improve the reproducibility of the work that we publish. This form provides structure for consistency and transparency in reporting. For further information on Nature Portfolio policies, see our Editorial Policies and the Editorial Policy Checklist.

Statistics

For all statistical analyses, confirm that the following items are present in the figure legend, table legend, main text, or Methods section.

n/a	Confirmed	
☐	☒	The exact sample size (*n*) for each experimental group/condition, given as a discrete number and unit of measurement
☐	☒	A statement on whether measurements were taken from distinct samples or whether the same sample was measured repeatedly
☐	☒	The statistical test(s) used AND whether they are one- or two-sided<br>*Only common tests should be described solely by name; describe more complex techniques in the Methods section.*
☒	☐	A description of all covariates tested
☒	☐	A description of any assumptions or corrections, such as tests of normality and adjustment for multiple comparisons
☐	☒	A full description of the statistical parameters including central tendency (e.g. means) or other basic estimates (e.g. regression coefficient) AND variation (e.g. standard deviation) or associated estimates of uncertainty (e.g. confidence intervals)
☐	☒	For null hypothesis testing, the test statistic (e.g. *F*, *t*, *r*) with confidence intervals, effect sizes, degrees of freedom and *P* value noted<br>*Give P values as exact values whenever suitable.*
☒	☐	For Bayesian analysis, information on the choice of priors and Markov chain Monte Carlo settings
☒	☐	For hierarchical and complex designs, identification of the appropriate level for tests and full reporting of outcomes
☒	☐	Estimates of effect sizes (e.g. Cohen's *d*, Pearson's *r*), indicating how they were calculated

Our web collection on statistics for biologists contains articles on many of the points above.

Software and code

Policy information about availability of computer code

Data collection	Gen5 program (version 3.10) from Bio-Tek for acquisition of cell images; Deltavision PersonelDV microscope (Applied Precision Inc., Issaquah, WA) for acquisition of autophagy and aggrephagy images; iBright Imaging systems (ThermoFisher Scientific) for quantitative western blots; QuantStudio real-time PCR system (ThermoFisher Scientific) for acquisition of qPCR data.
Data analysis	ImageJ (version 1.51) for Western blot quantitation; Prism 6 (Version 6.0d, GraphPad Software) for statistical analysis and graph presentation; SoftWoRx version 6.1.3. (GE Healthcare) for cell image analysis; Microsoft Excel (version 16.54) for qPCR data analysis.

For manuscripts utilizing custom algorithms or software that are central to the research but not yet described in published literature, software must be made available to editors and reviewers. We strongly encourage code deposition in a community repository (e.g. GitHub). See the Nature Portfolio guidelines for submitting code & software for further information.

Data

Policy information about availability of data

All manuscripts must include a data availability statement. This statement should provide the following information, where applicable:

- Accession codes, unique identifiers, or web links for publicly available datasets
- A description of any restrictions on data availability
- For clinical datasets or third party data, please ensure that the statement adheres to our policy

All the data that support the conclusions in this paper are available within this article and its supplementary Information file. Source data are provided with this paper.

Human research participants

Policy information about studies involving human research participants and Sex and Gender in Research.

Reporting on sex and gender	Not applicable
Population characteristics	Not applicable
Recruitment	Not applicable
Ethics oversight	Not applicable

Note that full information on the approval of the study protocol must also be provided in the manuscript.

Field-specific reporting

Please select the one below that is the best fit for your research. If you are not sure, read the appropriate sections before making your selection.

☒ Life sciences ☐ Behavioural & social sciences ☐ Ecological, evolutionary & environmental sciences

For a reference copy of the document with all sections, see nature.com/documents/nr-reporting-summary-flat.pdf

Life sciences study design

All studies must disclose on these points even when the disclosure is negative.

Sample size	The statistical significance was based on the statistical analysis method described in the paper. The sample sizes were chosen based on our prior research outcomes and experience, which guided us with a minimum N number to obtain statistically significant difference between samples (PMID: 25533187; 27046250; 29313410; 31208283). Two-tailed Student t-test was used with $p < 0.05$ for statistical significance.
Data exclusions	No data were excluded except when experiments were judged to be improperly conducted based on criteria used as control.
Replication	To validate and confirm the presented results, we conducted multiple repetitions of the same experiments. These repetitions occurred at different intervals, ranging from several weeks to months, and even a few years for some experiments. We used multiple different cell lines and similar types of treatments to further confirm the presented results. The statistical analysis described in the paper served as a measure of reproducibility.
Randomization	This is not applicable for all the experiments except cell imaging studies. To avoid any bias in taking cell images, we chose discrete sets of image areas in the same positions of each plate from different samples.
Blinding	This is not applicable for all the experiments except cell imaging studies. To avoid any bias in taking cell images, we used alphabetic codes for slides whose samples can be identified only after images are taken or swapped samples in the dark room where the imaging system is located so not to identify the order of samples taken during the imaging experiments.

Reporting for specific materials, systems and methods

We require information from authors about some types of materials, experimental systems and methods used in many studies. Here, indicate whether each material, system or method listed is relevant to your study. If you are not sure if a list item applies to your research, read the appropriate section before selecting a response.

Materials & experimental systems

n/a	Involved in the study
☐	☒ Antibodies
☐	☒ Eukaryotic cell lines
☒	☐ Palaeontology and archaeology
☐	☒ Animals and other organisms
☒	☐ Clinical data
☒	☐ Dual use research of concern

Methods

n/a	Involved in the study
☒	☐ ChIP-seq
☒	☐ Flow cytometry
☒	☐ MRI-based neuroimaging

Antibodies

Antibodies used

Antibodies and chemicals used in the experiments were obtained from the following sources: antibodies for ULK1 (sc-10900 for immunoprecipitation (IP) and sc-33182 for western blotting (WB) of human protein), Atg14 (sc-164767 for IP), Beclin 1 (sc-11427 for WB and sc-10086 for IP), p62 (sc-28359), and GAPDH (sc-25778) from Santa Cruz Biotechnology; anti-ULK1 antibody (A7481 for WB of mouse protein), anti-WIPI2 antibody (SAB4200400 for immunostaining), Earle's Balanced Salt Solution (EBSS) (2888), Bafilomycin A1 (B1793-10UG), blasticidin S (SBR00022-1ML), doxycycline hyclate (D9891-1G), polybrene (107689), oligomycin A (75351), antimycin A (A8674), and CCCP (C2759) from Sigma-Aldrich; A769662 (A3963-50), rotenone (B5462), MG132 (A2585), MRT-68921 (B6174-5), SAR405 (A8883-2) and Z-VAD-FMK (A1902-1) from ApexBio (Houston, TX); 991 (S8654) and GSK621 (S7898) from Selleckchem (Houston, TX); MK8722 (HY-111363) from MedChem Express (Monmouth Junction, NJ); Hydroxychloroquine (HCQ, 26301-0250) from Acros Organics (Geel, Belgium); antibodies for Vps34 (3358), AMPK (2532), Atg7 (8558), LKB1 (3047), Atg9a (13509), Atg14 (rabbit monoclonal clone D3H2Z for immunostaining and WB), mTOR (2972 and 2983 for WB), phospho-ACC (3661), phospho-Akt Ser473 (4051), Akt (9272), LC3B (2775 for WB), phospho-S6K1 Thr389 (9205), S6K1 (9202), phospho-Atg13 Ser355 (isoform 2 Ser318; mouse Ser354) (26839), phospho-ULK1 Ser758 (6888), phospho-ULK1 Ser556 (5869), phospho-ULK1 Ser317 (37762), phospho-ULK1 Ser638 (14205), cleaved PARP (9541), cleaved caspase-3 (9661) from Cell Signaling Technology; William's medium E (W4128), Active recombinant AMPKα1/β1/γ1 complex (14-840), anti-myc 9E10 monoclonal antibody (OP10), anti-ubiquitin antibody (04-263), and WIPI2 antibody (MABC91 for immunostaining) from EMD-Millipore; anti-HA antibody HA.11 from Covance (AFC-101P); LC3B antibody (PM036 for immunostaining) and p62 antibody (PM045 for immunostaining) from MBL International Corporation (Woburn, MA); Hanks' balanced salt solution (HBSS, 14175095), Lipofectamine 3000 (L3000015), puromycin (A11138-03), hygromycin B (10687010), anti-rabbit IgG (H+L) (A16029), anti-mouse IgG (H+L) (A16078), anti-goat IgG (H +L) (A16005), Alexa Flour 488-conjugated anti-rabbit IgG (A-21441), Alexa Flour 555-conjugated anti-mouse IgG (A-31570), and TRIzol Reagent (15-596-018) from ThermoFisher Scientific; Torin 1 (4247) and rapamycin (1292/1) from R&D; anti-phosphatidylinositol (840042P) from Avanti Polar Lipids; PI3P Grip (G-0302) from Echelon Biosciences; Protein G-agarose bead (P9202) from GenDEPOT; polyvinylidene difluoride membrane from Bio-Rad (1620177); Dulbecco's Modified Eagle's Medium (DMEM, 25-500) and horseradish peroxidase substrate ProSignal Pico kit from Genesee Scientific (20-300B). Recombinant N-terminal 10xHis-tagged Atg14 was obtained from Escherichia coli as a custom order to MyBioSource (San Diego, CA). Anti-Atg13 antibodies are described in our previous report (PMID: 19225151). Anti-pT660 antibody was made using PRNR(pT)LPDL-C as an antigenic peptide in rabbits, and purified using antigenic peptide-conjugated column in Abclonal Science, Inc. (Woburn, MA). Antibodies for pAtg14 Ser29 and pBeclin 1 Ser30 were previously described (PMID: 27046250; 29313410).

Validation

The primary antibodies used for immunoprecipitation (IP) and western blotting (WB) have been validated as described. Unless otherwise specified, the antibodies have been validated and utilized for WB of both mouse and human proteins. When the antibodies were used for IP or immunostaining, it was explicitly noted. Primary antibody binding was performed at 1:1000 dilution following the manufacture's recommendation. The dilution of the antibodies for immunostaining (1:100) was indicated explicitly in Methods section.

- ULK1 (sc-10900 for IP and sc-33182 for WB of human protein), Atg14 (sc-164767 for IP), Beclin 1 (sc-11427 for western blotting (WB) and sc-10086 for IP), p62 (sc-28359), and GAPDH (sc-25778) from Santa Cruz Biotechnology have been validated in our previous reports (PMID: 19225151; 25533187; 27046250; 29313410; 31208283).

- ULK1 (A7481 for WB of mouse protein) and WIPI2 (SAB4200400 for immunostaining) from Sigma-Aldrich have been validated in our previous reports (PMID: 27046250; 29313410; 31208283).

- Vps34 (3358), Atg7 (8558), Atg9a (13509), Atg14 (rabbit monoclonal clone D3H2Z for immunostaining and WB), mTOR (2972 and 2983 for WB), phospho-Akt Ser473 (4051), Akt (9272), LC3B (2775 for WB), phospho-S6K1 Thr389 (9205), S6K1 (9202), and phospho-Atg13 Ser355 (isoform 2 Ser318; mouse Ser354) (26839) from Cell Signaling Technology have been validated in our previous reports (PMID: 12150925; 19225151; 25533187; 27046250; 29313410; 31208283).

- AMPK (2532), LKB1 (3047), phospho-ACC (3661), phospho-ULK1 Ser758 (6888), phospho-ULK1 Ser556 (5869), phospho-ULK1 Ser317 (37762), phospho-ULK1 Ser638 (14205), cleaved PARP (9541), and cleaved caspase-3 (9661) from Cell Signaling Technology have been validated by the manufacturer. The validation data are available on the company's website (http://www.cellsignal.com).

- Myc 9E10 monoclonal antibody (OP10) and WIPI2 antibody (MABC91 for immunostaining) from EMD-Millipore have been validated in our previous reports (PMID: 12150925; 19225151; 25533187; 27046250; 29313410; 31208283).

- Ubiquitin antibody (04-263) from EMD-Millipore has been validated by the manufacturer. The validation data are available on the company's website (https://www.emdmillipore.com).

- HA antibody HA.11 from Covance (AFC-101P) from Enzo Life Sciences has been validated in our previous reports (PMID: 12150925; 19225151; 25533187; 27046250; 29313410; 31208283).

- LC3B antibody (PM036 for immunostaining) and p62 antibody (PM045 for immunostaining) from MBL International Corporation (Woburn, MA) have been validated in our previous reports (PMID: 27046250; 29313410; 31208283) for LC3B and by the manufacturer for p62 (https://www.mblbio.com/bio/g/).

- Anti-Atg13 antibodies are described in our previous report for the validation (PMID: 19225151).

- Anti-pT660 antibody was made using PRNR(pT)LPDL-C as an antigenic peptide in rabbits, and purified using antigenic peptide-conjugated column in Abclonal Science, Inc. (Woburn, MA). The antibody was validated in the current paper.

- Antibodies for pAtg14 Ser29 and pBeclin 1 Ser30, the sites we identified, were previously described in our reports for the validation (PMID: 27046250; 29313410).

Eukaryotic cell lines

Policy information about cell lines and Sex and Gender in Research

Cell line source(s)	HCT116 (CCL-247), HeLa (CCL-2), HEK293T (CRL-11268), HepG2 (HB-8065), C2C12 (CRL-1772), and A549 cells (CCL-185) were obtained from ATCC. HT22 cells (SCC129) were obtained from EMD-Millipore. 293FT cells were obtained from ThermoFisher Scientific. The ULK DKO MEFs are from Dr. Mondira Kundu; the AMPK DKO MEFs are from Dr. Benoit Viollet; the LKB1 KO MEFs are from N. Bardeesy; the Atg9A KO MEFs are from S. Akira and T. Saitoh. All other knockout cell lines and stably-transduced cell lines described in this study have been developed by the corresponding author's laboratory.
Authentication	The original cell lines used in this study were authenticated by the suppliers, based on analysis of genome sequences such as short tandem repeats, isoenzyme analysis, DNA fingerprinting, and/or karyotyping. The knockout cell lines and stably-transduced cells, which we generated, were validated for every experiment, in which they were used, by genotyping, western blotting, and/or DNA sequencing.
Mycoplasma contamination	All cell lines were confirmed to be mycoplasma-free using MycoAlert PLUS Mycoplasma Detection kit (Lonza Walkersville, Inc. LT07).
Commonly misidentified lines (See ICLAC register)	No commonly misidentified cell lines were used in the study.

Animals and other research organisms

Policy information about studies involving animals; ARRIVE guidelines recommended for reporting animal research, and Sex and Gender in Research

Laboratory animals	C57BL6 male mice were purchased from the Jackson Laboratory. Mice between 10 and 12 weeks of age were used for the experiment.
Wild animals	No wild animals were used in the study.
Reporting on sex	The study used only male mice. We have included this information in the manuscript. The study did not involve sex-based analyses because the main purpose of the analysis was to determine acute effects of drugs in autophagy, which is considered not critically affected by sex difference. The study was to confirm cell culture data.
Field-collected samples	No field collected samples were used in the study.
Ethics oversight	The experimental procedures were approved by the University of Minnesota, Institutional Animal Care and Use Committee.

Note that full information on the approval of the study protocol must also be provided in the manuscript.
